# Age‐related changes in fluid biomarkers and cognitive abilities in a non‐human primate model of AD pathology

**DOI:** 10.1002/alz70856_105057

**Published:** 2026-01-07

**Authors:** Akash G Patel, Pramod N Nehete, Noor Salaymeh, Bharti P Nehete, Sohail Karimi, Ludovic Debure, Qiao Zhang, Fengzhu Wang, Lisa M Pytka, Yongzhao Shao, Michele Marie Mulholland, William D Hopkins, Thomas Wisniewski, Henrieta Scholtzova

**Affiliations:** ^1^ NYU Grossman School of Medicine, New York, NY, USA; ^2^ UT MD Anderson Cancer Center, Michale E. Keeling Center for Comparative Medicine and Research, Bastrop, TX, USA; ^3^ New York University Langone Health, New York, NY, USA; ^4^ New York University Grossman School of Medicine, New York, NY, USA

## Abstract

**Background:**

Current immunotherapeutic approaches for Alzheimer's disease (AD) are limited in their effectiveness and face complications associated with cerebral amyloid angiopathy (CAA), such as amyloid‐related imaging abnormalities (ARIA). Our earlier published work using a TLR9 agonist, CpG‐ODN, to modulate the innate immune system for treating AD, has benefited from the use of non‐human primates (NHP), squirrel monkeys (SQMs), which naturally develops AD pathology, including extensive age‐dependent CAA. In this study, we further the development of this model by characterizing trajectories of biofluid biomarkers and cognitive changes across the SQM lifespan, the dynamics that have never been described before.

**Method:**

Plasma and CSF biomarkers of AD pathogenesis (Ab), neurodegeneration (NfL, GFAP, neurogranin), cerebrovascular dysfunction (MMPs), and inflammation (YKL‐40/CHI3L1, sTREM2, S100B) were characterized cross‐sectionally using SIMOA and Luminex immunoassays. New age cohorts were supplemented into our dataset. A touchscreen‐based Automated Cognitive Testing System (ACTS) was implemented within SQM cohorts to assess behavioral performance.

**Result:**

Our current work represents continuation of our comprehensive cross‐sectional study on aging and CAA/AD pathogenesis in SQMs. By applying simple linear regression models, we observe age‐associated decreases in plasma Aβ40, Aβ42, and Aβ42/Aβ40 ratio. Additionally, CSF levels of NfL, GFAP, and plasma YKL‐40, sTrem2 demonstrated significant linear increases across disease continuum. Segmented regression analyses revealed changepoints in CSF Aβ40, Aβ42, Aβ42/Aβ40 ratio and plasma MMP3, that were followed by a significant decrease in slope. Plasma concentrations of NfL, GFAP, neurogranin, and S100B significantly increased with age after the estimated changepoint. We are further exploring SQM fluid biomarker profiles using the NULISAseq platform. Moreover, ACTS Transfer Index Task revealed that geriatric SQMs perform significantly worse than young SQMs in the learning phase, while there were no differences in the reversal trials. In the Conceptual Set Shifting Task, we found a difference in learning latency between young and geriatric SQMs. Ongoing efforts are focused on integrating fluid biomarkers with cognitive and neuroimaging measures.

**Conclusion:**

We believe that successful incorporation of this NHP model will improve diagnostic capability and mechanistic understanding of AD pathology development, particularly CAA, as well as help quicken the progress towards new therapeutics with an assessment of their associated ARIA risk.

